# BMC Surgery reviewer acknowledgement, 2014

**DOI:** 10.1186/1471-2482-15-8

**Published:** 2015-02-13

**Authors:** Guangde Tu

**Affiliations:** BioMed Central, Room 1006-1007 Financial Plaza, No. 333 Jiujiang Road, Huangpu District, 200001 Shanghai China

## Abstract

The editors of BMC Surgery would like to thank all our reviewers who have contributed their time to the journal in Volume 14 (2014).

## Contributing reviewers

**Tamer Abdelbaki**

Egypt

**Thad Abrams**

USA

**Walid Abu Arab**

Canada

**Tomohiro Adachi**

Japan

**Mohsen Adib-Hajbaghery**

Iran

**Sunil Agarwal**

India

**Rajesh Aggarwal**

United Kingdom

**Magnus Ågren**

Denmark

**Ferdinando Agresta**

Italy

**Annette B. Ahrberg**

Germany

**Clemens Aigner**

Austria

**Sami Akbulut**

Turkey

**Melih Akinci**

Turkey

**Luca Antonio Aldrighetti**

Italy

**Marco Allaix**

Italy

**William Allum**

United Kingdom

**Emad Aly**

United Kingdom

**Roland Andersson**

Sweden

**Martin Angele**

Germany

**Stavros Antoniou**

Greece

**Saif Anwaruddin**

USA

**Theerachai Apivatthakakul**

Thailand

**Asif Ayaz**

India

**Egemen Ayhan**

Turkey

**Nather Aziz**

Singapore

**Marcus Bahra**

Germany

**Seung Hyuk Baik**

South Korea

**Gian Luca Baiocchi**

Italy

**Andreas Bakoyiannis**

Greece

**Carlo Banfi**

Switzerland

**Gordon Bannister**

United Kingdom

**Nikolaos Barbetakis**

Greece

**Louise Barbier**

France

**Irene Bargellini**

Italy

**Neal Barshes**

USA

**Giacomo Batignani**

Italy

**Hasan Batirel**

Turkey

**Dirk Bausch**

Germany

**Jens Bedke**

Germany

**Marcelo Beltran**

Chile

**Christophe Berney**

Australia

**Frederik Berrevoet**

Belgium

**Samer Bessa**

Egypt

**Ashish Bhalla**

India

**Deepraj Bhandarkar**

India

**Roland Biber**

Germany

**Franck Billmann**

Germany

**Marcel Binnebosel**

Germany

**Desmond Birkett**

USA

**Alessandro Boellis**

Italy

**Marja Boermeester**

Netherlands

**Stefano Bogliolo**

Italy

**Therezia Bokor**

Germany

**Patrick Boland**

USA

**Giuseppe Borzellino**

Italy

**Sotirios Botaitis**

Greece

**Sergiu Botolin**

USA

**Mahdi Bouassida**

Tunisia

**Michael Brauckhoff**

Norway

**Marius Bredell**

Switzerland

**Stefan Breitenstein**

Switzerland

**Brandon Broome**

USA

**Merribeth Bruntz**

USA

**Jacek Budzynski**

Poland

**Orhan Bulut**

Denmark

**Jakob Burcharth**

Denmark

**Neslihan Cabioglu**

Turkey

**Michael Calderwood**

USA

**Fabio Campanile**

Italy

**Giampiero Capobianco**

Italy

**Fernando Carbonell-Tatay**

Spain

**Cédric Carrié**

France

**Ross Carruthers**

United Kingdom

**Lucas Castro Alves**

Brazil

**Juan Cata**

USA

**Fausto Catena**

Italy

**Abigail Caudle**

USA

**Marco Ceresoli**

Italy

**Hakan Ceyran**

Turkey

**Nohra Chalouhi**

USA

**Shi-Min Chang**

China

**Grigoris Chatzimavroudis**

Greece

**Gar-Yang Chau**

Taiwan

**Yen-Lin Chen**

Taiwan

**Michael Chen**

Taiwan

**Wei Chen**

China

**Yong-Long Chi**

China

**Jy Ming Chiang**

Taiwan

**Sonja Chiappetta**

Germany

**Vitoon Chinswangwatanakul**

Thailand

**Charing Chong**

Hong Kong

**Giovanni Conzo**

Italy

**Riccardo Corbetta**

Italy

**Andrea Saverio Corriero**

Italy

**Wilson Costa**

Brazil

**Marie Crandall**

USA

**Derly Cuellar**

USA

**Steven Cunningham**

USA

**Adrien Daigeler**

Germany

**Piergiorgio Danelli**

Italy

**Marcioi De Moraes**

Brazil

**Belinda De Simone**

Italy

**Faramarz Dehghani**

Germany

**Ismail Demiryilmaz**

Turkey

**Timm Denecke**

Germany

**Jacopo Desiderio**

Italy

**Andrew Deynichenko**

Ukraine

**Salomone Di Saverio**

Italy

**Gaetano Di Vita**

Italy

**Marius Distler**

Germany

**Cornelia Dotzenrath**

Germany

**Michael Douek**

United Kingdom

**Jianjun Du**

China

**Xavier Ducrocq**

France

**Ender Dulundu**

Turkey

**Traian Dumitrascu**

Romania

**Gülsüm Özlem Elpek**

Turkey

**Tobias Else**

Germany

**Xiaoen Hua**

China

**Itaru Endo**

Japan

**Giorgio Ercolani**

Italy

**Mert Erkan**

Germany

**Unal Erkorkmaz**

Turkey

**Eloy Espin**

Spain

**Khashayar Fakhrian**

Germany

**Mazda Farshad**

Switzerland

**Denis Fedorov**

Ukraine

**Israel Fernandez-Pineda**

Spain

**Pedro Ferreira**

Portugal

**Enrico Fiori**

Italy

**Jochen Franke**

Germany

**Oren Fruchter**

Israel

**Sean Galvin**

Australia

**Shu-Guang Gao**

China

**Cengiz Gebitekin**

Turkey

**Ian Geh**

United Kingdom

**Dechun Geng**

China

**Pascal Gervaz**

Switzerland

**Wagih Ghnnam**

Egypt

**Peter Giannoudis**

United Kingdom

**Luca Gianotti**

Italy

**Sonja Gillen**

Germany

**Montori Giulia**

Italy

**Gabriel Glockzin**

Germany

**Stanislaw Gluszek**

Poland

**Dhanny Gomez**

United Kingdom

**Jackson A. Gondim**

Brazil

**Shouping Gong**

China

**Matthias Goos**

Germany

**Ajeet Gordhan**

USA

**Fred Gordon**

USA

**Rosalie Grivell**

Australia

**Stephen Grobmyer**

USA

**Jodok Matthias Grueneberger**

Germany

**Dirk Grünhagen**

Netherlands

**Salvatore Gruttadauria**

Italy

**Oleg Gulko**

Ukraine

**Ali Guner**

Turkey

**Subhash Gupta**

India

**Rajesh Gupta**

India

**Bora Gurer**

Turkey

**Thilo Hackert**

Germany

**David Hak**

USA

**Niels Hammer**

Germany

**Eric Mark Hammerberg**

USA

**Jiandong Hao**

USA

**Jonathan Hardman**

United Kingdom

**Wolfgang Hartschuh**

Germany

**Hassan Hassan**

USA

**Arvid Steinar Haugen**

Norway

**Alexander Hemprich**

Germany

**Ralf Henkelmann**

Germany

**Lars Henningsohn**

Sweden

**Benoit Herbert**

USA

**Noriyuki Hirahara**

Japan

**Andreas Höch**

Germany

**Jens Hoeppner**

Germany

**Werner Hohenberger**

Germany

**Mathias Hoiczyk**

Germany

**Peter Hollaus**

Germany

**Ulrich Theodor Hopt**

Germany

**Zhiyong Hou**

China

**Yoshiyuki Hoya**

Japan

**Karel WE Hulsewe**

Netherlands

**Igors Iesalnieks**

Germany

**Abdolmajid Iloon Kashkouli**

Iran

**Daniela Ionescu**

Romania

**Mitsuru Ishizuka**

Japan

**Hiroaki Ito**

Japan

**Kimieru Ito**

Japan

**Shinji Itoh**

Japan

**Usman Jaffer**

United Kingdom

**Sandeep Jain**

India

**Priyadarshan Jategaonkar**

India

**Kuo-Shyang Jeng**

Taiwan

**Jack Jia**

China

**Jorge Jiménez Cruz**

Germany

**George Jour**

USA

**Kethy Jules-elysee**

USA

**Francis Juthier**

France

**Daniel Kaemmerer**

Germany

**Sahin N Kahramanca**

Turkey

**Adrien Kaladji**

France

**Erdinc Kamer**

Turkey

**Kenitiro Kaneko**

Japan

**Oguz Karahan**

Turkey

**Cemşit Karakurt**

Turkey

**Samuel Andreas Käser**

Germany

**Ozgur Kemik**

Turkey

**Jeong Han Kim**

South Korea

**Ji Wan Kim**

South Korea

**Eugene Kim**

USA

**Young-Woo Kim**

South Korea

**Hirokazu Kiyozaki**

Japan

**Christian Daniel Klink**

Germany

**Akira Kobayashi**

Japan

**Kiyotaka Kohshi**

Japan

**Arto Kokkola**

Finland

**Eun-jung Kong**

South Korea

**Manousos Konstadoulakis**

Greece

**Kostiantyn Kopchak**

Ukraine

**George Kouvelos**

Greece

**Bela Kubat**

Netherlands

**Henry Mark Kuerer**

USA

**Simon Kuesters**

Germany

**Vinay Kumaran**

India

**Anita Kurmann**

Switzerland

**Miltiadis Lalountas**

Greece

**Vincent Lam**

Australia

**Felix Langer**

Austria

**Hryhoriy Lapshyn**

Germany

**Bashir Laway**

India

**Morgan LE Guen**

France

**Dong Hoon Lee**

South Korea

**Maw-Sheng Lee**

Taiwan

**Han Hong Lee**

South Korea

**Woo Jung Lee**

South Korea

**Kuno Lehmann**

Switzerland

**Ray Leveillee**

USA

**François L’Hériteau**

France

**Laura Lorenzon**

Italy

**Tatiana Lubenets**

Ukraine

**David Lumenta**

Austria

**Xiaofeng Lv**

China

**Vladimir Lyadov**

Russian Federation

**Giovanni Maconi**

Italy

**Antonio Macrì**

Italy

**Almantas Maleckas**

Lithuania

**Luigi Marano**

Italy

**Rafik Margaryan**

Italy

**Goran Marjanovic**

Germany

**Stephen Mark**

New Zealand

**Lukas Marti**

Switzerland

**Shigeki Matsubara**

Japan

**Ippei Matsumoto**

Japan

**Blandine Maurel**

France

**Amine Mazine**

Canada

**Craig McIlhenny**

United Kingdom

**Ruth McKee**

United Kingdom

**Sarah McLaughlin**

USA

**Ari N. Meguerditchian**

Canada

**Anastasia Mentessidou**

Greece

**Aziz Merchant**

USA

**Juerg Metzger**

Switzerland

**Yutaka Midorikawa**

Japan

**Radu Mihai**

United Kingdom

**Marco Milone**

Italy

**Aytan Miranda Sipahi**

Brazil

**Petros Mirilas**

USA

**Yevgen Miroshnychenko**

Ukraine

**Suhail Mithani**

USA

**Teruaki Mizobuchi**

Japan

**Irwin Mohan**

Australia

**Yasuhiko Mohri**

Japan

**Michele Molinari**

Canada

**Yvonne Mondorf**

Germany

**Francisco L Moreno Martinez**

Cuba

**Martin Mortazavi**

USA

**Ahmet Yaser Muslumanoglu**

Turkey

**Miki Nagao**

Japan

**Toru Nagao**

Japan

**Myura Nagendran**

United Kingdom

**Takeshi Naitoh**

Japan

**Yoshifumi Nakao**

Japan

**Margherita Nannini**

Italy

**Steven Narod**

Canada

**Atsushi Nashimoto**

Japan

**Christian Andreas Nebiker**

Switzerland

**Valentin Neuhaus**

Switzerland

**Michael Norwood**

United Kingdom

**Alexander Novotny**

Germany

**Dagmar Oberhofer**

Croatia

**Jooyoung Oh**

South Korea

**Lawrence Okiror**

United Kingdom

**Peter O'Leary**

Ireland

**Terje Osnes**

Norway

**Hannu Paajanen**

Finland

**Andrea Pakula**

USA

**Norbert Pallua**

Germany

**Stefano Palmucci**

Italy

**Basilios Papaziogas**

Greece

**Ji Hoon Park**

South Korea

**Jeong Yoon Park**

South Korea

**Vani Parmar**

India

**Jennifer Paruch**

USA

**Marta Pascual**

Spain

**Andrea Patrizi**

France

**Milos Pavlovic**

Slovenia

**Florentia Peintinger**

Austria

**Erman Pektok**

Turkey

**Gianluca Pellino**

Italy

**Nadia Peparini**

Italy

**Tirso Perez-Medina**

Spain

**Giuseppe Piccinni**

Italy

**Laurentiu Pirtea**

Romania

**John Plukker**

Netherlands

**Elia Poiasina**

Italy

**Daniel Prevedello**

USA

**Pablo Priego**

Spain

**Borja Quiroga**

Spain

**Juan C Quispe**

USA

**Nikolina Radulovich**

Canada

**Marco Raffaelli**

Italy

**Beate Rau**

Germany

**Stefano Rausei**

Italy

**Asnat Raziel**

Israel

**Matthias Reeh**

Germany

**Joachim Reibetanz**

Germany

**Philipp Renner**

Germany

**Mehmet Reyhan**

Turkey

**Paul RISS**

Austria

**Andreas J. Roth**

Germany

**Andres Mariano Rubiano Escobar**

Colombia

**Muhammad Saaiq**

Pakistan

**Anand Sachithanandan**

Malaysia

**Babak Saedi**

Iran

**Mauro Salizzoni**

Italy

**Paulina Salminen**

Finland

**Ozgur Samancilar**

Turkey

**Mohamed Samra**

Egypt

**Andrés Sánchez-Pernaute**

Spain

**Gabriel Sandblom**

Sweden

**Massimo Sartelli**

Italy

**Kirsten Sasaki**

USA

**Atsushi Sasaki**

Japan

**Jens Schittenhelm**

Germany

**Rick Schneider**

Germany

**Paulus Schurr**

Germany

**Andreas Seim**

Norway

**Raffaele Serra**

Italy

**Giuseppe Filiberto Serraino**

Italy

**Piergiorgio Settembrini**

Italy

**Yan-Shen Shan**

Taiwan

**Aali Sheen**

United Kingdom

**Minxin Shi**

China

**Chikashi Shibata**

Japan

**ChungLiang Shih**

Taiwan

**Satoshi Shiono**

Japan

**Robert Siegel**

Germany

**Sumit Sinha**

India

**Silvia Siqueira**

Brazil

**Anatolii Skums**

Ukraine

**Nicholas Slater**

Netherlands

**Matthew Smith**

United Kingdom

**Jonathan Sobocinski**

France

**Salvatore Sorrenti**

Italy

**Michael Sosin**

USA

**Cristiano Spadaccio**

Belgium

**Constantine Spanos**

Greece

**Ulrich Spiegl**

Germany

**Kai Sprengel**

Switzerland

**Andrew Sprowson**

United Kingdom

**Michael Stenger**

Denmark

**Dominika Stygar**

Poland

**Juan Manuel Suarez Grau**

Spain

**Hasan Sunar**

Turkey

**Andrew Swift**

United Kingdom

**Oleg Symonov**

Ukraine

**Tomasz Szewczyk**

Poland

**Yuji Tachimori**

Japan

**Frank Tacke**

Germany

**Arash Taheri**

USA

**Hidenori Takahashi**

Japan

**Shinsuke Takeno**

Japan

**Mohammad Talebpour**

Iran

**Dinesh Talwar**

United Kingdom

**Carol Tan**

United Kingdom

**Kuniya Tanaka**

Japan

**Mikhail Tavobilov**

Russian Federation

**Konstantinos Tepetes**

Greece

**Yuji Toiyama**

Japan

**Aybala Tongut**

Turkey

**Christos Tourmousoglou**

Greece

**Vincent Traynelis**

USA

**Gary Tse**

Hong Kong

**Akif Turna**

Turkey

**Garth Utter**

USA

**Todd Vander Heiden**

USA

**Horatiu Vasian**

Romania

**Mohammad Vaziri**

Iran

**Ali Veral**

Turkey

**Andras Vereczkei**

Hungary

**Jorrit Jan Verlaan**

Netherlands

**Nereo Vettoretto**

Italy

**Ishwarappa Balekundri Vijayalakshmi**

India

**Charles Vincent**

United Kingdom

**Minutolo Vincenzo**

Italy

**Brendan Visser**

USA

**Ernst von Dobschuetz**

Germany

**Rüdiger M O Von Eisenhart-Rothe**

Germany

**Urs Von Holzen**

Switzerland

**Nicolas H. von der Hoeh**

Germany

**Michael Wall**

Australia

**Yuedong Wang**

China

**Atsushi Watanabe**

Japan

**David Watters**

Australia

**Lizzy Weigelt**

Switzerland

**Ulrich Friedrich Wellner**

Germany

**Sandy Widder**

Canada

**Philipp Wiggermann**

Germany

**Allison Williams**

USA

**Donna Williams**

USA

**Uwe Wittel**

Germany

**Reuben Wong**

Singapore

**Chaoneng Wu**

China

**Jianwei Xie**

China

**Katsuhiko Yanaga**

Japan

**Hee Chul Yang**

South Korea

**Chun-Nan Yeh**

Taiwan

**Erdal Yekeler**

Turkey

**Christopher Young**

Australia

**Takafumi Yukaya**

Japan

**Giovanni Zagli**

Italy

**Dirk Zajonz**

Germany

**Denise Zantut Wittmann**

Brazil

**Kamran Zargar Shoshtari**

New Zealand

**Sergii Zemskov**

Ukraine

**Enver Zerem**

Bosnia and Herzegovina

**Yingze Zhang**

China

**Chunfeng Zhao**

USA

**Jiawei Zheng**

China

**Cheng Zhou**

China

**Xuesong Zhu**

China

**Olexiy Zubkov**

Ukraine

